# Artificial intelligence integrated WeChat social media adoption for collaborative learning engagement among university students

**DOI:** 10.1038/s41598-026-40611-6

**Published:** 2026-03-17

**Authors:** Isaac Kofi Mensah, Muhammad Khalil Khan

**Affiliations:** 1 School of Accounting, Wuhan College, Wuhan, Hubei People’s Republic of China; 2 Department of Journalism and Communication, School of Media and Law, NingboTech University, Ningbo, People’s Republic of China; 3 Belt and Road International Communication Research Center, NingboTech University, Ningbo, People’s Republic of China

**Keywords:** Social media, Artificial intelligence, Collaborative learning, Resource sharing, Moderating influence, University students, Technology acceptance model, WeChat, Business and management, Business and management, Education, Information systems and information technology, Science, technology and society

## Abstract

Artificial intelligence (AI) has transformed social media into a powerful tool for educational learning engagements. WeChat, a Chinese social media platform, is extensively used by students for academic learning, collaboration, and information acquisition. However, research on the adoption of AI-integrated social media for learning engagement remains limited, especially in the Chinese context. Based on the traditional Technology Acceptance Model (TAM), this study proposes an AI-Integrated Social Media Adoption (AISMA) model for learning engagement by integrating the core TAM constructs—perceived usefulness (PU) and perceived ease of use (PEOU), with the external factors of collaborative learning, social support, resource sharing, and facilitating conditions to investigate their influence on students’ intention to adopt AI-integrated WeChat for learning engagement. Data from Chinese university students were analyzed using structural equation modeling (SEM) via Smart-PLS 4. The results confirm that collaborative learning, social support, and resource sharing are significant direct drivers of AI-integrated social media adoption. Furthermore, facilitating conditions significantly influence both PU and PEOU and also directly drive adoption intention. A key finding from the moderating analysis is that PEOU significantly amplifies the effects of all three external factors (collaborative learning, social support, and resource sharing) on adoption intention. While PU significantly strengthens the relationship for social support and resource sharing, it does not moderate the link between collaborative learning and adoption intention. The study concludes by discussing the theoretical contributions and practical implications for educators and platform developers.

## Introduction

The rise of large language models (LLMs) and Artificial Intelligence (AI) has significantly altered the operational methods of social media^1^. The utilization of AI in social media is expanding at an unparalleled rate, distinctly reshaping the framework of social media platforms^2^. AI integration in social media (SM) platforms such as WeChat is transforming the education sector in China. Scholars believe that AI and social media have become a transformative agent in the modern educational environment, and AI-integrated technologies are positively shaping students’ academic performance and well-being^3^. SM is a digital technological system that permits the sharing of ideas and information based on user-generated content or interaction in formats such as texts and visuals via virtual systems and societies^4,5^. SM use in education has transformed the traditional concept of education by integrating technology to drive educational access and bringing effectiveness and efficiency in teaching systems^6,7^. SM systems empower higher learning institutions to transform traditional face-to-face-oriented education with virtual teaching and learning via SM for sustainable education^8,9^. SM has become the most effective way to create a sustainable formal teaching atmosphere for students^8^. SM is also reshaping teaching methods and access to learning resources, and significantly increasing students’ active participation in the classroom^10^. SM provides students with a two-way communication channel, enabling them to engage in diversified exchanges and achieve more efficient real-time collaboration^7,10^. The ubiquity of social media and its societal impact have revolutionized the way people share knowledge, communicate, and collaborate^11^.

Furthermore, social media has evolved into a key resource for supporting students’ academic performance and improving their grades, significantly enhancing their participation and educational effectiveness by deeply integrating into the learning process^12,13^. It also provides students with a new perspective on knowledge acquisition, enabling them to engage in both formal learning (such as course content) and informal learning (such as independently searching for key information) at the same time^12^. Through these approaches, students can meet like-minded people and engage in in-depth knowledge exchanges by sharing their own learning experiences^14,15^. The importance of SM for students stems from its openness and global reach, which allows them to connect, share information, and build networks without being limited by time and location^16,17^. Besides, Universities are integrating social media into education to empower students through better communication, information exchange, and collaborative content creation^18^. Social media’s key advantages in education are improved communication, enhanced peer collaboration, quick resource sharing, accessible materials, and the development of career-relevant tech skills^19,20^.

AI integration in social media enhances instructional strategies to cater to the needs of students and personalized learning, creating a more engaging and exciting learning environment^21^. AI-integrated social media is an advanced interactive platform that empowers students to share thoughts and ideas with peers and collaboratively work together on projects^21^. AI-integrated social media also provides unique means to bridge educational inequalities in underprivileged regions and societies by facilitating equal access to education and enhancing performance^22^. Therefore, the integration of SM with AI offers a meaningful and engaging learning experience in areas that involve a variety of learning tasks and facilitate productive communications among peers^23^. AI integration in education-linked applications, such as AI-integrated social media (AISM), stimulates creativity, enhances engagement through interactive elements, and provides personalized feedback that supports emotional well-being^24^.

This research article aims to explore Chinese university students’ intention to adopt AI-integrated SM (WeChat-–Yuanbao) for learning collaboration and engagement. The study extends the existing Technology Acceptance Model (TAM) to incorporate collaborative learning, resource sharing, social support, and facilitating conditions in the model. AI advancements are creating new paradigms for student development, revolutionizing educational methodologies and social interaction through personalized content, enhanced collaboration, and more efficient communication^1^. AI empowers students to actively reflect on information they get from social media^25^. For instance, after watching multiple reels on a specific topic, students can ask AI to generate a summary. This helps them consolidate the information and gain a deeper understanding of the material^25^. AI assists students in synthesizing key insights from video and social media content, while also monitoring their overall learning progression^25^. While the importance of AI-integrated social media has significantly transformed students’ learning engagement, there is, however, a deficit of studies that examine students’ adoption behavior of AI-integrated social media for collaborative learning engagement, particularly from the Chinese context.

Research shows that when students learn collaboratively and share resources, they find social media more useful and easier to use, which makes them more likely to adopt it for learning^26,27^. While perceived usefulness (PU) and perceived ease of use (PEOU) are known to be important, it is unclear how they specifically affect the way collaborative learning, social support, and resource sharing lead to the adoption of AI-integrated platforms like WeChat in China. This study addresses this gap by exploring the role of PU and PEOU in Chinese students’ intention to use AI-integrated WeChat for better learning engagement. TAM is considered an authoritative and useful framework for explaining the use of new technological systems and social media in education^27,28^. Therefore, TAM is fully consistent with the goals and objectives set by this study, specifically: first, to evaluate the influence of collaborative learning, social support, resource sharing, and facilitating conditions on the adoption intention of AI-integrated SM among university students. Second, to assess the impact of facilitating conditions on PU and PEOU of AI-integrated SM among university students, and thirdly, to analyze the moderating effects of PU and PEOU on the relationship between collaborative learning, social support, resource sharing, and students’ adoption intention of AI-integrated SM. Based on these objectives, the following research questions are formulated:

RQ1: How do collaborative learning, social support, resource sharing, and facilitating conditions stimulate the adoption intention of AI-integrated SM (WeChat-Yuanbao) among university students for learning engagement?

RQ2: How do facilitating conditions impact the PU and PEOU of AI-integrated SM (WeChat-Yuanbao) among students for learning engagement?

RQ3: How do PU and PEOU, respectively, moderate the impact of collaborative learning, social support, and resource sharing on students’ adoption intention of AI-integrated SM (WeChat-Yuanbao) for learning engagement?

Investigating these research questions offers practical policy implications for policy-makers, educationists, and key stakeholders, including educational institutions, to devise strategic systems to harness the usage of AI-integrated SM for effective information and learning engagements among students to achieve enhanced learning and better academic performance.

## Background of the study

### WeChat—Yuanbao

China has emerged as the largest SM industry, with a vast number of active users globally^29^. China’s social platforms are leading the next wave of AI, deploying advanced features that redefine interaction, content creation, and digital engagement^30^. From personalized feeds to AI-assisted creation tools, China’s social platforms have evolved into intelligent ecosystems. WeChat, Douyin (TikTok), Xiaohongshu (RedNote), and other Chinese social media platforms are employing AI not just as a feature, but as the core engine for captivating and seamless user experience^30^. WeChat is one of the most prevalent SM in China’s digital environment^29^. It is the predominant mobile communication and social media platform in mainland China, facilitating instantaneous text- and voice-mediated dialogues^31^. The platform (WeChat) has further evolved into a central tool for cultivating interpersonal networks and sustaining social ties, thereby becoming deeply embedded in users’ digital lifestyles^32,33^. China’s technology conglomerate, Tencent, developed WeChat in 2011. WeChat, locally known as Weixin, was upgraded with a new AI-assistant app called Yuanbao (元宝) in early 2025^34^. This integration of the AI-assistant app—Yuanboa into WeChat (WeChat – Yuanbao) empowers more than one billion users in China to interact with AI services without downloading a separate app^34^. Now, WeChat users can add Yuanbao as a “friend” and start a conversation directly within the app^34^. With AI-powered capabilities for search, summarization, and writing, Yuanbao can analyze complex documents and engage in dynamic, prompt-driven conversations^35^. The formal integration of Yuanbao into the WeChat ecosystem, with its goal of becoming a personal companion for over 1.3 billion users, signals a pivotal change in the platform’s strategic direction^36^. The integration of the AI assistant ‘Yuanbao’ into WeChat, China’s largest social platform, signals a major shift, making AI a foundational infrastructure^37^. By embedding it directly into WeChat, Tencent is leveraging its vast ecosystem to gain a decisive advantage in the AI field^37^.

### WeChat—Yuanbao and collaborating learning engagement

WeChat—Yuanbao is powered by Tencent’s own large language model (LLM)—Hunyaun, and it functions as both a standalone application (Yuanbao—元宝) as well as a deeply embedded feature in WeChat. It works as an intelligent assistant to support collaborative learning by providing instant explanations, processing group information, managing collective tasks, sharing insights, and retrieving knowledge on demand. It gives vital cognitive support to maintain effective group problem-solving capabilities and alleviate learners’ frustration. Furthermore, it can facilitate collaborative document summarization/analysis and provide multilingual translation capabilities to support the co-creation of a shared understanding and lower communication barriers within diverse learning teams. Therefore, Yuanbao is not merely an add-on AI feature but is deeply embedded into the WeChat ecosystem, which has the potential to deliver tailored content and act as an effective collaborative learning agent ^38^. Its adoption and use represent a concrete mechanism through which AI integration in social media transforms collaborative learning engagement and perceived learning outcomes. This showcases AI’s power to transform engagement and create a more inclusive education system^39^. AI-driven recommendations power the WeChat-Yuanbao, connecting learners who have complementary skills to collaborate and exchange knowledge^40^.

## Theoretical framework and research model

### Technology acceptance model (TAM)

The Technology Acceptance Model (TAM), based on the Theory of Reasoned Action, explains the fundamental mechanisms between an individual user’s internal beliefs, attitudes, and behavioral intentions. It can predict and explain people’s adoption behavior of information technology-mediated systems Ajzen^41^. This study chose this model because it has been widely validated, is simple and robust, and can reliably explain and predict individuals’ acceptance of information systems. TAM believes that the actual use of technology depends on individual behavioral intentions^42^. The willingness to use new technologies is driven by both an individual’s attitude toward the technology and their perception of the system’s effectiveness^42^. The attitude is projected to be driven by two cardinal constructs, such as PU and PEOU^42^. Additionally, PEOU is considered to indirectly influence the intention to use via PU^42,43^. Overall, the TAM put forward that the most important drivers of technology adoption are PU (i.e., the level of confidence individuals have in the idea that employing a specific system will improve their job productivity) and PEOU (i.e., an understanding that utilizing a particular information system will require no effort) of a technological system. Amongst the factors considered in the TAM, the PU construct is considered to be the strongest driver of an information system use intention^44^.

TAM has been used frequently to handle research problems relating to social media adoption and usage among students^45^. Some of the frequent constructs that have been extended with TAM include perceived trust and security, perceived connectedness, perceived enjoyment, subjective norms, perceived critical mass, and self-efficacy^45^. Also, a systematic review of TAM showed that the majority of the research works using TAM were undertaken in higher educational institution settings^45^.

### AI-integrated social media adoption for collaborative learning engagement

AI-integrated social media refers to the integration of AI technologies into social media platforms that enhance the user experience and streamline content management to improve platforms’ utilization, effectiveness, and efficiency for learning engagement. It is not just about platforms using AI in their back-end as a recommendation engine instead of offering users distinct, interactive AI-driven features as a core part of the social media interface and functionality^46^. AI-integrated social media has become a pivotal factor of collaborative learning engagement and is now deeply embedded in the native social ecosystem. Consequently, our extension of TAM with specific variables (Collaborative Learning, Social Support, and Resource Sharing) is theoretically warranted to empower us to theorize the factors that emerge at the intersection or integration of AI technology and social media systems. Additionally, the inclusion of constructs such as collaborative learning, social support, and resource sharing to extend TAM is based on the following theoretical justification, as these variables are fundamental social behaviors, already tested in social media systems^47^:

- Collaborative Learning: In standard social media, collaboration is organic but unstructured. In this context, the AI acts as an embedded facilitator^47^, facilitating team formations and prompting focused discussion threads. This transforms casual social interaction into a structured and goal-oriented collaboration learning without leaving the built-in platform^48^, such as WeChat—Yuanbao.
- Social Support: Social media inherently provides peer support^49^. The integration of an AI assistant amplifies this by offering immediate, personalized cognitive and affective support (e.g., the WeChat—Yuanbao Assistant providing instant explanations). This creates a dual-layer support system—an AI-supported immediate aid, and a human peer support^50^ to reduce frustration and sustain engagement within the same social interface.
- Resource Sharing: Social media enables link- and file-sharing^51,52^. The integration of AI in social media transforms this into intelligent, proactive curation by analyzing group discussions and individual queries. AI Assistants such as WeChat—Yuanbao can push context-relevant resources (articles, videos, tools) directly into the chat or feed, making resource sharing dynamic, personalized, and timely, turning the social stream into a learning-centric content network.

Additionally, in the extended TAM, we position PU and PEOU as major moderators in our model as compared to the direct (linear antecedents to intention) validated effect of these variables on social media and AI technology adoption intention^53,54^. In a platform where social and learning activities are intertwined, perceptions of usefulness and ease of use are not just initial adoption triggers but ongoing filters. We hypothesize that PU and PEOU moderate the impact of our key variables because, first, if users already find the AI useful (high PU), AI-facilitated collaborations will feel more productive, strengthening the link between collaborative learning and sustained intention. Secondly, if the AI feels seamlessly integrated into the familiar social media interface, such as WeChat (high PEOU), the barrier to seeking AI-mediated support or resources drops, enhancing its effect. In essence, we argue that PU and PEOU determine whether the AI’s social learning features are perceived as natural enhancements or as disruptive complexities. Consequently, our extension moves TAM toward a context-specific theory rather than a universal theory.

### Research model

**Fig. 1:**
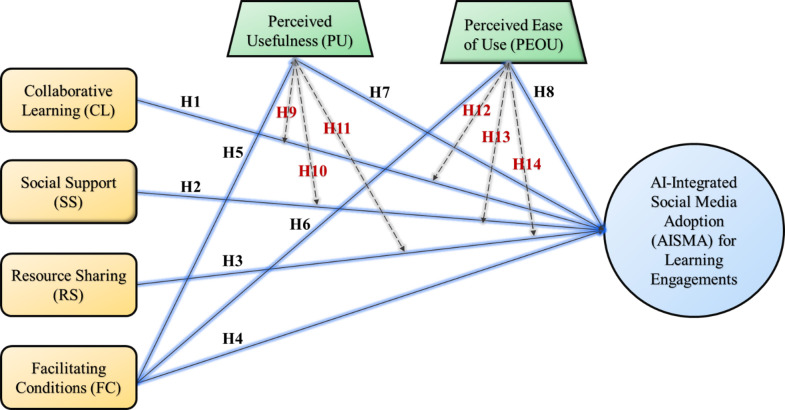
Proposed conceptual research model.

Our research model, as demonstrated in Fig. 1, proposes that collaborative learning, social support, resource sharing, facilitating conditions, PU, and PEOU will positively influence AI-integrated WeChat adoption intention. In addition, FC will influence PU and PEOU. At the same time, PU and PEOU will act as moderators to moderate the influence of collaborative learning, social support, and resource sharing on AI-integrated WeChat adoption intention.

## Research hypotheses

### Collaborative learning

Collaborative learning is a learning procedure. It ensures that two or more individuals come together to develop meaning, discuss a matter, or sharpen their skill sets^55^. Collaboration is a vibrant value of communication and a personal lifestyle characterized by individual accountability, where individuals recognize and appreciate the abilities and contributions of others while also taking responsibility for their actions^56^. An educational style of teaching and collaborative learning has to do with groups of learners working in unison to tackle challenges, complete tasks, or product creation^56^. Technologies such as SM have been created to drive and encourage collaborative learning engagements for learning and knowledge development^55^. Thus, collaborative learning through the SM describes how students engage with their peers through SM platforms, classmates, friends, and teachers to achieve collaborative learning goals^27^. SM has transformed the nature of communication and collaborative learning space for learners^27^. SM is essential in community development, improving collaboration and communication between SM users^57^. Furthermore, SM has been projected to improve student academic empowerment and performance via student learning programs and team communication^58^.

SM can also be used to enhance the four-categorized benefits of collaborative learning: (a) social benefits—build a social support mechanism for students, advance learning communities, and boost diverse understanding between students and teachers; (b) psychological benefits—enhance learners’ self-confidence, reduce anxiety due to cooperation, and build positive attitudes towards teachers; (c) academic benefits— inspires critical thinking, promote students’ active learning, enhance performance, and improves problem-solving skills; (d) assessment benefits—use teaching modes for different assessments^56^. Therefore, the use of AI-integrated SM to achieve these four major benefits of collaborative learning engagements might drive students’ intentions to use SM for collaborative learning. Recent research has also demonstrated that collaborative learning is positively connected with SM adoption^27,59^. Accordingly, H1 was proposed.

H1: Collaborative learning will positively influence students’ intention to adopt AI-integrated social media (WeChat) for learning engagement.

### Social support

Social support is a mechanism-based perception in which people seek help from their supportive social networks—friends, family, and classmates, etc^60,61^. Social support can take various forms to assist individuals, including emotional support, which involves validating personal feelings; informational support, which provides advice; companionship, which fosters a sense of belonging; and instrumental support, such as financial assistance^62^. Each of these social support functions is instrumental in maintaining and developing people’s positive mental well-being^62^. There are three major functions of social support from a communication standpoint, i.e., informational support, interpersonal social support—positive social interaction, and emotional support^63,64^. The information social support provides needs-based counseling, information, and direction, or a response that offers assistance in solving problems^65^. Interpersonal social support functions to provide a positive social interaction, which includes spending quality periods with others during relaxation, and it can also be in the form of sharing experiences with others in times of challenges^65^. Lastly, emotional support ensures there is love, acceptance, caring, and encouragement among social support networks^62^. Social support is also visible in online settings via SM (i.e., online social support), which empowers individuals, including students, to get peer support (interpersonal support) via interacting with SM friends^62,66^. In addition, online social support via SM gives critical informal support in the mode of comradeship, including emotional support and tangible aid^67^. In fact, the primary goal of any person’s social network is to offer social support for its members^67^. Consequently, online friendship, emotional support, and tangible aid that students can get from virtual SM can enhance their learning outcomes, which might drive their adoption of SM. Therefore, SM can become an essential social support tool for students’ personal development and academic performance during their studies. Research has confirmed that SM use among students can augment their perception of various SM support during their stay on campus (adjustment to college environment)^68^. Accordingly, H2 was proposed.

H2: Social support will positively influence students’ intention to adopt AI-integrated social media (WeChat) for learning engagement.

### Resource sharing

SM provides enormous opportunities for resource sharing and dissemination of user-generated content among users for educational and personal development. Resource sharing is the individual motivation to share their resources in terms of information, teaching, and learning materials with others via SM^69^. Thus, SM provides a collaborative setting to encourage knowledge-seeking, development, and sharing in learning and teaching contexts^26^. Resource sharing via SM offers students the unique chance to bond, share experiences and ideas, and develop their education horizons (i.e., knowledge development and sharing, a culture of cooperation, suppleness, and approachability, community building with shared domain and rich resources)^70^. AI-integrated SM enhances resource sharing, including knowledge, information, online community development, and supportive interactions, which might influence students’ willingness to adopt these platforms more broadly. Research has shown that resource sharing significantly drives the adoption of SM^26^. Consequently, H3 was projected.

H3: Resource sharing will positively influence students’ intention to adopt AI-integrated social media (WeChat) for learning engagement.

### Facilitating conditions

Facilitating conditions (FC) refer to the degree to which individuals recognize the availability of organizational and technological infrastructure that supports the use of a technological system^71^. These technological and organizational infrastructures are designed to eradicate barriers that may limit users from enjoying the maximum benefits from technology adoption^72^. FC can also be classified into two types of conditions: resource-facilitating conditions and technological-facilitating conditions^72^. Resource and technological facilitating conditions are both critical in encouraging SM adoption. Therefore, if suitable conditions exist for the usage of SM, e.g., good internet connectivity, availability of cheap mobile data and broadband, free Wi-Fi connections, affordable connecting devices such as mobile handsets, stable power supply, and swift technical support, then individuals would be more attracted to use such an information system^73,74^. Research confirms that FCs are significant drivers of the behavioral intention to use new innovative technologies^75,76^, if users perceive them as useful (PU)^77^, and easy to use (PEOU)^78^. Hence, H4, H5, and H6 were put forward.

H4: FC will positively influence students’ intention to adopt AI-integrated social media (WeChat) for learning engagement.

H5: FC will positively influence the PU of AI-integrated social media (WeChat) for learning engagement.

H6: FC will positively influence the PEOU of AI-integrated social media (WeChat) for learning engagement.

### Perceived usefulness

Perceived usefulness (PU) refers to the user’s satisfaction of enjoying the maximum benefits in utilizing a technological/information system. For example, the PU of the new technological systems will be higher if their utilization boosts a person’s job output^42^. PU is a major and stronger driver of technology adoption among people^42,43^. The usefulness of AI-integrated SM to empower students to efficiently utilize SM to achieve greater performance output in learning engagement activities will encourage its wider adoption among students. Research has shown that the PU of SM has a direct effect on social media adoption^79^. Accordingly, H7 was proposed.

H7: PU will positively influence students’ intention to adopt AI-integrated social media (WeChat) for learning engagement.

### Perceived ease of use

Perceived ease of use (PEOU) is a belief that the usage of a new innovative system is easier and effort-free to complete the task^42,43^. Therefore, it is considered a significant driver of technology adoption, as highlighted in TAM^42^. Numerous studies suggested that PEOU positively influences attitudes regarding the adoption of new technological systems like SM^26,78^. AI-integrated SM systems that are designed with features that enhance students’ usage experience will lead to better adoption. Research has validated that the PEOU of SM positively influences the adoption of SM^27^. Hence, we proposed that:

H8: PEOU will positively influence students’ intention to adopt AI-integrated social media (WeChat) for learning engagement.

### Moderating influence of perceived usefulness

The benefits that a technology provides can have multiplier effects on the adoption of such a technology, e.g., AI-integrated social media systems. In this study, it is proposed that the PU of AI-integrated SM can positively moderate the impact of collaborative learning, social support, and resource sharing on the adoption intention of AI-integrated SM. Consequently, we proposed H9, H10, and H11.

H9: PU of AI-integrated social media positively moderates the relationship between collaborative learning and students’ intention to adopt WeChat for learning engagement.

H10: PU of AI-integrated social media positively moderates the relationship between social support and students’ intention to adopt WeChat for learning engagement.

H11: PU of AI-integrated social media positively moderates the relationship between resource sharing and students’ intention to adopt WeChat for learning engagement.

### Moderating influence of perceived ease of use

The nature of technological systems’ design, such as AI-integrated social media systems that might place less burden on users to operate them, will have a magnifying effect on the adoption of such a technology. Previous studies confirmed that PEOU can mediate the influence of collaborative learning on SM adoption^26^. However, to further validate the impact of PEOU on SM adoption, this study proposes that PEOU potentially can also moderate the effect of collaborative learning, social support, and resource sharing on the students’ adoption intention of SM. Accordingly, H12, H13, and H14 were suggested.

H12: PEOU of AI-integrated social media positively moderates the relationship between collaborative learning and students’ intention to adopt WeChat for learning engagement.

H13: PEOU of AI-integrated social media positively moderates the relationship between social support and students’ intention to adopt WeChat for learning engagement.

H14: PEOU of AI-integrated social media positively moderates the relationship between resource sharing and students’ intention to adopt WeChat for learning engagement.

## Research methodology

### Questionnaire development

We utilize a quantitative research design to validate the model illustrated in Fig. 1. A well-designed research questionnaire was prepared to gather data for analysis. The questionnaire constructs were adopted from prior works and were modified to fit the context of this study. We adopted the construct items from the following sources: collaborative learning^26,80^, social support^62^, resource sharing^26,81^, facilitating conditions^82^, PU^42,43^, PEOU^42,43^, and behavioral adoption intention^83^. Each of these constructs comprised three questions, which were assessed using a five-point Likert scale, where 1 represented strong disagreement, and 5 indicated strong agreement. The questionnaire was organized into two distinct parts: the first part presented information concerning the model shown in Fig. 1, and the second part collected essential demographic data from the respondents, such as their age, gender, educational background, etc. The research items and their reliability indicators are shown in Table 2.

To facilitate a comprehensive understanding of the questionnaire by the respondents whose primary language of instruction was Chinese, the questionnaire was translated from English into Chinese and back-translated. Translation and back-translation were undertaken by two experts in the fields of social media adoption and education who were conversant in English and Chinese. Back translation verified that the translated version remained faithful to the original meaning^84^. To further ensure quality data collection processes, the questionnaire instrument was pre-tested and piloted with fifty (50) university students. Pre-testing and piloting are carried out to evaluate the questionnaire and survey processes to determine if they pose any challenges to the respondents^85,86^. The pre-testing and piloting phases allowed researchers to critically evaluate the efficiency of the questionnaire/scales, assessing their content validity, the clarity and sequence of questions, and other aspects of questionnaire design, including layout, formatting, typography, and overall length.

### Sampling technique and data collection

The convenience and snowball sampling method using social media was employed to gather responses from the participants, as it provides easy access to respondents at very low costs^87^. The snowball sampling approach was utilized to amplify the effectiveness of convenience sampling through snowball’s referral system, where study subjects recruit future subjects from their colleagues, friends, or acquaintances^88,89^. Thus, participants were encouraged to refer and share the questionnaire with their classmates and friends in their respective universities to reach a wider student population. Ultimately, while convenient sampling guaranteed easy access to participants, the snowball method empowered the study to recruit more participants via other participants^89^. The questionnaire was hosted online at https://www.wjx.cn/, and the link, as well as the QR code, was shared through the WeChat platform to target students from different Universities in Central China, Wuhan, Hubei Province. SM was utilized since it offers readily available respondents who are frequently on social media and thus provides an easier means of data collection. The ethical approval (No. NTU-IRB25-SML13) of the study was granted by the Institutional Review Board (IRB) of NingboTech University, Ningbo, China. This study strictly abides by the institutional and 2024 Declaration of Helsinki ethical guidelines. An informed consent form was obtained from all the respondents along with the questionnaire. Participants were made aware that their participation in the survey was entirely voluntary and that they had the option to withdraw from the survey at any point. The questionnaire link and QR code were distributed between May and June 2025. A total of 440 valid questionnaires were collected, and the data were subsequently analyzed using Smart PLS Software.

### Smart PLS-SEM as the analytical approach

We used Partial Least Squares Structural Equation Model (PLS-SEM) to test the hypothesis and validate the research model. PLS-SEM combines the advantages of prediction and theoretical verification, and is particularly suitable for analyzing sophisticated structural models with numerous variables and complex relationships^90^. In addition, it can help researchers to deeply explore the theoretical extensions of existing theories and promote exploratory research with theoretical construction value^90^. Therefore, it is the preferred tool for this study due to its flexibility and ease of use, as well as its powerful analytical capabilities.

## Data analysis and results

### Demographic information

From the collected samples, there were 150 males, accounting for 34.1%, and 290 females, accounting for 65.9% of the total sample. The rest of the sample information is summarized in Table 1.

**Table 1:** Respondent demographic information.

	Frequency	Percentage
**Gender**		
Male	150	34.1
Female	290	65.9
**Age range**		
18–25 years	345	78.4
26–30 years	78	17.7
31–35 years	17	3.9
**Current education**		
Bachelor’s degree	329	74.8
Master’s degree	70	15.9
Ph.D.	41	9.3
**Social media usage experience**		
1–5 years	110	25.0
6–10 years	226	51.4
11+	104	23.6

### Common method bias (CMB)

We used the Harman single-factor process and EFA (exploratory factor analysis) to detect issues of CMB. The results showed that the unrotated first factor explained only 27.980% of the variance, well below the 50% warning threshold, indicating that the data in this study were free of significant common method bias.

### Measurement model

We employed Cronbach’s Alpha (CA) and composite reliability (CR) metrics to assess the reliability and internal consistency of the scales. Scholars^91^ suggest that scales possess a satisfactory level of internal consistency if the CA and CR values of the scale exceed the standard threshold of 0.70. Table 2 indicates that our scale values for CA and CR, for each construct, are above the required threshold of 0.70. Hence, each scale achieved internal consistency.

The convergent validity (CV) of the scale was measured through factor loadings (FL), CR, and average variance extracted (AVE). The results in Table 2 indicate that the FL, CR, and AVE values of each scale exceeded the required thresholds of 0.6 for FL, 0.7 for CR, and 0.5 for AVE^90,92^. Therefore, all the scales used in this study met the convergent validity standard.

To ensure that the concepts in the model are truly independent of each other, we used two well-established methods: the Fornell-Larcker criterion and the HTMT ratio. The Fornell-Larcker criterion compares a construct’s square root of the AVE with its correlation coefficient to its relationships with others. A construct’s higher square root of the AVE indicates that the construct is sufficiently independent and not confounded with others. Table 3 indicates that the square root of the AVE of all constructs is higher than their correlation with other constructs. This is an indication that discriminant validity has been achieved^93^. Additionally, the HTMT analysis (see Table 4) further confirmed that the HTMT values of all the constructs are well below the required value of 0.85, indicating the strong discriminant validity of the model^90^. Consequently, the model demonstrates good discriminant validity.

**Table 2:** Scale reliability and convergent validity.

Variables	Items	Factor loading	Cronbach’s alpha	CR	AVE
Collaborative learning (CL)	**CL1**: The AI features on WeChat (Yuanbao for smart search, translation, study bots, etc.) help my study groups share knowledge and develop new skills more effectively.	0.880	0.869	0.920	0.792
	**CL2**: Using AI-powered tools within WeChat (Yuanbao for generating summaries or problem- solving) makes collaborative learning with my peers more efficient and productive.	0.886			
	**CL3**: AI-enhanced communication on WeChat (Yuanbao for AI-assisted translation in group chats or smart replies) facilitates smoother interaction with classmates and teachers for learning purposes.	0.905			
Social support (SS)	**SS1**: I use AI features on WeChat (Yaunbao for fun filters, games, or chatbots) to connect with others for relaxation and comfort.	0.863	0.837	0.902	0.754
	**SS2**: In times of challenge, I can use WeChat’s AI tools (Yuanbao for smart search or information) to quickly find helpful advice and resources, supported by my friends and family on the platform.	0.881			
	**SS3**: The AI-driven communities and recommendation systems on WeChat help me find specialized online support groups that I might not have discovered in my offline life.	0.861			
Resource sharing (RS)	**RS1**: Using AI tools on WeChat (Yuanbao for translation or content generators) helps me share knowledge and resources with others more effectively.	0.885	0.847	0.907	0.766
	**RS2**: The process of creating and sharing resources is more enjoyable and efficient when I use WeChat’s AI-powered features (Yuanbao AI-assistant for editing or formatting).	0.880			
	**RS3**: Sharing knowledge through AI features on WeChat (Yuanbao for explaining a concept with an AI-generated diagram) helps me deepen my own understanding of the subject.	0.860			
Facilitating conditions (FC)	**FC1**: I have the necessary support (e.g., from my university or peers) to help me use Yuanbao AI-Assistant on WeChat for my learning.	0.873	0.828	0.897	0.744
	**FC2**: The internet connectivity (Wi-Fi, mobile data) is available and reliable enough to smoothly use the data-intensive Yuanbao AI-Assistant on WeChat for real-time translation and AI search, etc.	0.861			
	**FC3**: The cost of data and internet access is affordable for me to regularly use WeChat’s AI tools (Yuanbao) for learning activities.	0.853			
Perceived usefulness (PU)	**PU1**: The AI features on WeChat (Yuanbao for smart search, translation, study aids) are useful for my studies and research.	0.910	0.880	0.925	0.805
	**PU2**: Using WeChat’s AI tools (Yuanbao) helps me complete my academic tasks more quickly.	0.903			
	**PU3**: Using Yuanbao AI-Assistant on WeChat enhances my overall learning productivity.	0.878			
Perceived ease of use (PEOU)	**PEOU1**: Interacting with the AI features on WeChat (Yuanbao for smart search, or AI assistants) is clear and understandable.	0.864	0.840	0.903	0.755
	**PEOU2**: It is easy for me to learn how to use WeChat’s AI tools (Yuanbao) effectively.	0.856			
	**PEOU3**: I find WeChat’s AI-powered functions easy to operate.	0.888			
AI-integrated social media adoption (AISMA)	**AISMA1**: I intend to use AI-integrated features (Yuanbao) on WeChat for my learning activities.	0.883	0.836	0.901	0.753
	**AISMA2**: I plan to regularly use AI-integrated features (Yuanbao) on WeChat as a part of my learning process.	0.866			
	**AISMA3**: I recommend my peers to use AI-integrated features (Yuanbao) on WeChat for learning.	0.854			

**Table 3:** Discriminant validity-Fornell-Larcker criterion.

Variables	CL	SS	RS	FC	PU	PEOU	AISMA
CL	**0.890**						
SS	0.386	**0.868**					
RS	0.452	0.422	**0.875**				
FC	0.322	0.234	0.268	**0.863**			
PU	0.150	0.146	0.099	0.214	**0.897**		
PEOU	0.093	0.042	-0.046	0.237	0.325	**0.869**	
AISMA	0.346	0.344	0.424	0.376	0.109	0.220	**0.868**

CL=Collaborative Learning, SS=Social Support, RS=Resource Sharing, FC=Facilitating Conditions, PU=Perceived Usefulness, PEOU=Perceived Ease of Use, AISMA = AI-integrated Social Media Adoption.

**Table 4:** Discriminant validity-heterotrait-monotrait ratio (HTMT).

Variables CL	CL	SS	RS	FC	PU	PEOU	AISMA
SS	0.453	-					
RS	0.527	0.498	-				
FC	0.380	0.280	0.317	-			
PU	0.171	0.169	0.113	0.247	-		
PEOU	0.104	0.066	0.058	0.278	0.379	-	
AISMA	0.405	0.412	0.502	0.451	0.122	0.261	-

CL=Collaborative Learning, SS=Social Support, RS=Resource Sharing, FC=Facilitating Conditions, PU=Perceived Usefulness, PEOU=Perceived Ease of Use, AISMA = AI-integrated Social Media Adoption.

### Structural model

#### Multicollinearity analysis

This study uses the variance inflation factor (VIF) to test whether the model has any multicollinearity issues. Hair, Ringle^94^ argue that if the inner model VIF values are less than 5, it indicates that there is no multicollinearity problem in the model. Table 5 indicates that the highest VIF value within the inner model is 1.480 between RS and AISMA, which is far below the standard threshold value of 5. Hence, our model does not possess multicollinearity issues.

**Table 5:** Multicollinearity diagnosis—inner model matrix.

Variables	VIF		
	PU	PEOU	AISMA
CL			1.456
SS			1.375
RS			1.480
FC	1.000	1.000	1.291
PU			1.187
PEOU			1.200

CL=Collaborative Learning, SS=Social Support, RS=Resource Sharing, FC=Facilitating Conditions, PU=Perceived Usefulness, PEOU=Perceived Ease of Use, AISMA = AI-integrated Social Media Adoption.

#### Model explanation (R-square)

We used R-squared (R²) metrics (the coefficient of determination) to assess the explanatory power of our model. Chin^95^ argues that the explanatory power of the model is substantial if the R² value is equal to or above 0.67, and it is moderate if the R² value is equal to or above 0.33. The model possesses weak explanatory power if the R² value is equal to or below 0.19. Similarly, scholars (e.g.^90^, argue that if the R² is greater than zero, then it suggests that the model explains some portion of variance in the endogenous variable. Results in Table 6 indicate that the R² values for AISMA, PEOU, and PU are greater than zero. Furthermore, the coefficient of determination R² relative to AISMA reaches 0.513, meaning that the CL, SS, RS, and FC variables capture more than 50% of the variance of AISMA. This result gives the main AISMA model a clearly satisfactory explanatory power. Conversely, the R² observed for PEOU (0.056) and PU (0.046) remains very modest, revealing a limited explanatory capacity of these two dimensions.

**Table 6:** Model explanation (R-square).

	*R*-square	*R*-square adjusted
PU	0.046	0.044
PEOU	0.056	0.054
AISMA	0.513	0.499

PU=Perceived Usefulness, PEOU=Perceived Ease of Use, AISMA = AI-integrated Social Media Adoption.

#### Predicting relevance

We employed PLSpredict to assess the predictive relevance (Q^2^) of our model. Scholars^96^ noted that the model possesses significant predictive relevance and explanatory power if the value of Q^2^ exceeds zero. Results in Table 7 indicate that Q^2^ values for the PU (0.041), for PEOU (0.051), and for AISMA (0.218) are higher than the threshold value of zero. Hence, our model demonstrates effective predictive relevance.

**Table 7:** Predicting relevance (Q2) estimates.

Item	Q²predict
PU	0.041
PEOU	0.051
SMA	0.218

PU=Perceived Usefulness (PU), PEOU=Perceived Ease of Use, AISMA = AI-integrated Social Media Adoption.

### Hypothesis testing

#### Direct path coefficients

**Fig. 2:**
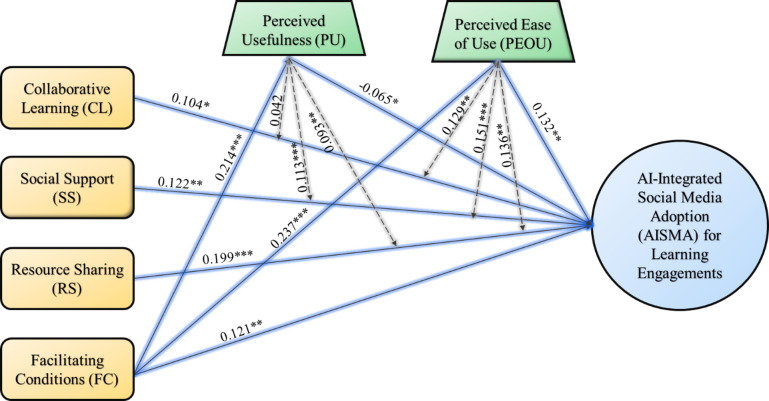
Validated Structural Equation Model of WeChat (AI-integrated Social Media Adoption).

We used SmartPLS 4.1.0 and performed bootstrapping with a 5000-subsample to estimate the path coefficient of the proposed model. Results in Table 8; Fig. 2 indicate that Collaborative Learning (CL) (β = 0.104, t = 2.242, *p*<0.05), Social Support (SS) (β = 0.122, t = 2.680, *p*<0.01), Resource Sharing (RS) (β = 0.199, t = 4.108, *p*<0.001), and Facilitating Conditions (FC) (β = 0.121, t = 2.951, *p*<0.01), have positively influenced the AI-integrated Social Media Adoption (AISMA) in China, whereas Facilitating Conditions (FC) also significantly affect the Perceived Usefulness (PU) (β = 0.214, t = 4.338, *p*<0.001), and Perceived Ease of Use (PEOU) (β = 0.237, t = 4.961, *p*<0.001) of AI-integrated Social Media. Hence, H1, H2, H3, H4, H5, and H6 are supported. In contrast to our proposed hypothesis, PU did not positively influence the AI-integrated Social Media Adoption (AISMA) (β=-0.065, t = 1.716, *p* < 0.05). However, PEOU was significant in driving the AI-integrated Social Media Adoption (AISMA) (β = 0.132, t = 2.814, *p* < 0.01). Hence, H7 was not supported while H8 was supported (See Table 8; Fig. 2).

**Table 8:** Hypothesis testing—direct path coefficient.

Hypothesis	Path	β	T statistics	*P* values	Results
H1	CL -> AISMA	0.104	2.242	0.013	Supported
H2	SS -> AISMA	0.122	2.680	0.004	Supported
H3	RS -> AISMA	0.199	4.108	0.000	Supported
H4	FC -> AISMA	0.121	2.951	0.002	Supported
H5	FC -> PU	0.214	4.338	0.000	Supported
H6	FC -> PEOU	0.237	4.961	0.000	Supported
H7	PU-> AISMA	-0.065	1.716	0.043	Not Supported
H8	PEOU-> AISMA	0.132	2.814	0.002	Supported

CL=Collaborative Learning, SS=Social Support, RS=Resource Sharing, FC=Facilitating Conditions, PU=Perceived Usefulness (PU), PEOU=Perceived Ease of Use, AISMA = AI-integrated Social Media Adoption.

#### Moderating effects

We also conducted moderation analysis using SmartPLS 4.1.0 with a 5000-bootstrapping sample to estimate the path parameters of the proposed model. Results in Table 8; Fig. 2 indicate that Perceived Usefulness (PU) significantly moderates the relationship between Social Support (SS) and AI-integrated Social Media Adoption (AISMA) (β = 0.133, t = 3.299, *p*<0.001) and between Resource Sharing (RS) and AI-integrated Social Media Adoption (AISMA) (β = 0.093, t = 2.134, *p*<0.05). However, PU fails to moderate the relationship between Collaborative Learning (CL) and AI-integrated Social Media Adoption (AISMA) (β = 0.042, t = 0.862, *p* > 0.05). Hence, H9 is rejected while H10 and H11 are supported (Table 9; Fig. 2).

**Table 9:** Moderation analysis.

Hypothesis	Path	β	T statistics	*P* values	Results
H9	PU x CL -> AISMA	0.042	0.862	0.194	Not supported
H10	PU x SS -> AISMA	0.133	3.299	0.000	Supported
H11	PU x RS -> AISMA	0.093	2.134	0.016	Supported
H12	PEOU x CL -> AISMA	0.129	2.452	0.007	Supported
H13	PEOU x SS -> AISMA	0.151	3.195	0.001	Supported
H14	PEOU x RS -> AISMA	0.136	2.810	0.002	Supported

CL= Collaborative Learning, SS= Social Support, RS= Resource Sharing, PU= Perceived Usefulness, PEOU=Perceived Ease of Use, AISMA = AI-integrated Social Media Adoption.

In addition, Perceived Ease of Use (PEOU) significantly moderates the relationship between Collaborative Learning (CL) and AI-integrated Social Media Adoption (AISMA) (β = 0.129, t = 2.452, *p* > 0.01), between Social Support (SS) and AI-integrated Social Media Adoption (AISMA) (β = 0.151, t = 3.195, *p*<0.001), and between Resource Sharing (RS) and AI-integrated Social Media Adoption (AISMA) (β = 0.136, t = 2.810, *p*<0.01). Therefore, H12, H13 and H14 are supported (Table 9; Fig. 2).

## Discussions with practical implications

This study utilizes an extended TAM to investigate the factors influencing students’ intention to adopt AI-integrated WeChat Social Media for collaborative learning engagement. The research model integrated the core TAM constructs of PU and PEOU with the external variables of collaborative learning, social support, resource sharing, and facilitating conditions. A key focus was to examine the moderating roles of PU and PEOU on the relationships between these external factors and adoption intention. The findings reveal that collaborative learning, social support, and resource sharing are all significant direct determinants of students’ intention to use AI-integrated social media for learning engagement. This underscores the importance of the platform’s capacity to enable peer interaction, provide emotional and informational support, and facilitate the exchange of academic resources. Furthermore, facilitating conditions were found to be a foundational driver, significantly enhancing both the perceived usefulness and ease of use of the AI tools, while also exerting a direct positive influence on adoption intention itself.

The moderating analysis indicates that PEOU significantly strengthened the positive effect of all three external factors—collaborative learning, social support, and resource sharing—on adoption intention. This suggests that when AI features are easy to interact with, they seamlessly amplify the benefits of collaboration, support, and sharing. Conversely, while PU also served as a significant moderator for social support and resource sharing, it did not significantly moderate the relationship between collaborative learning and adoption intention. This implies that the direct motivation to collaborate with peers is so strong that it does not rely on a heightened perception of the tool’s usefulness to drive adoption intention.

The significant influence of collaborative learning on adoption intention **(H1)** reveals that students are motivated to use AI-integrated social media when it enables them to cooperate in achieving shared academic goals^38^. This environment allows students to showcase their abilities, accept responsibility, and build consensus, providing strong social, psychological, and academic benefits. By facilitating communication beyond the classroom, these platforms become powerful resources for collaborative learning, fostering higher achievement, productivity, and supportive relationships^97^. Ultimately, this creates a supportive environment that encourages students to adopt AI-integrated social media not merely for socializing but as an essential tool for active, collaborative education that prepares them for real-world challenges. This finding backs studies that showed that collaborative learning is positively linked to SM adoption^26,27^. However, the finding contradicts a recent study^98^ that indicates collaborative learning does not significantly influence social media engagement.

The significant positive influence of social support on adoption intention **(H2)** highlights that students are driven to adopt AI-integrated social media when it serves as a reliable source of psychological and academic support. This encompasses the reciprocal exchange of information, constructive feedback, and encouraging interactions within the network. Such support reduces learning anxiety, enhances self-efficacy, and creates a supportive environment that empowers students to engage with these technologies, thereby fostering greater adoption intention. Our finding supports previous studies that social support is directly related to the adoption of SM^99^.

Furthermore, the significant influence of resource sharing on adoption intention **(H3)** underscores its role as a key utility of academic social media. Students are motivated to adopt social media platforms that enable the efficient, low-cost exchange of diverse learning materials, which directly enhances academic performance, growth, and well-being^3,97^. Ultimately, resource sharing cultivates an atmosphere of community and collaboration, which significantly amplifies adoption intention. This not only enriches the educational experience but also prepares students for the collaborative dynamics of their future careers. Our result aligns with studies that resource sharing on SM drives its adoption^26,100^. This also confirmed that information sharing is a critical component of resource sharing on social media, positively impacting students’ social media engagement^98^.

Additionally, the significant impact of facilitating conditions on both PU **(H5)** and PEOU **(H6)** demonstrates that institutional support is foundational to adoption. When universities provide reliable technological infrastructure, students are better able to appreciate the benefits and user-friendliness of AI-integrated social media for learning engagement. Furthermore, these conditions also exert a direct and significant influence on adoption intention itself **(H4)**, underscoring their fundamental role. Ultimately, by providing necessary resources and training, facilitating conditions not only enhance positive perceptions but also directly drive students’ intention to incorporate AI-integrated platforms into their educational activities. The results of this research corroborate previous studies indicating that facilitating conditions directly influence the PU and PEOU of SM systems^77^. It further endorses research indicating that enabling facilitating conditions affect the PEOU of SM systems^78^.

Moreover, the analysis yielded a counterintuitive yet statistically significant finding regarding PU. Contrary to conventional expectations and decades of TAM research, PU demonstrated a significant but negative relationship with AI-integrated Social Media Adoption (AISMA) (β = -0.065, *p* < 0.05) **(H7).** These findings contradict the traditional TAM research that posits the increase in PU resulted in higher adoption of technology. However, our study indicates that for every unit increase in a user’s belief that the AI-integrated social media tool would enhance their learning performance, there was a corresponding slight decrease in their actual adoption of it. Therefore, based on these results, PU acted as a weak but significant deterrent rather than a driver of adoption for the AI-integrated social media platform in the learning context. This suggests that other, potentially unmeasured, factors may have overshadowed the tool’s perceived benefits, leading to a paradoxical effect where increased perceived utility was associated with lower usage. This counterintuitive finding can be understood through the lens of the privacy paradox^101,102^ and innovation resistance^103,104^. Although adoption of AI-integrated social media for collaborative learning can be regarded as highly “useful,” such as automatic progress tracking, AI-matched grouping, personalized content recommendations, or AI tutoring systems, but it also raises serious concerns about individual privacy and data security, particularly in the context of China’s stringent data management policy and netizens’ growing awareness about privacy issues in China. The user may be more concerned about surveillance, data misuse, and the loss of learning autonomy than about its potential learning benefits. Thus, the “utility value” of these intelligent features may provoke psychological resistance or privacy concerns as the perceived risks attached with technology outweigh the functional benefits. Consequently, it might trigger negative spillover on the individuals’ willingness to adopt that technology. The perceived complexity of using AI-integrated social media may be another important factor for its lower adoption for collaborative learning. Therefore, the negative relationship between PU and adoption of AI-integrated social media for collaborative learning can be contextualized within the framework of privacy paradox^101,102^ and innovation resistance^103,104^ to understand the perceived usefulness of AI-integrated social media as a benefit or unnecessary risk for collaborative learning among Chinese students. Our results contradict previous studies that the perceived usefulness of social media systems is positive and significantly influences e-learning social media usage behavior^105^. It also differs from a study that the performance expectancy (usefulness) of AI is positively significant in driving AI adoption intention^106^.

Furthermore, the analysis revealed that PEOU emerged as a significant and positive driver of AI-integrated Social Media Adoption for learning engagement **(H8).** This finding, supported by a beta coefficient of 0.132 and a highly significant p-value of 0.002, indicates that the more students perceive these AI-integrated platforms as intuitive and effortless to use, the more likely they are to adopt them for their learning. The strength of this statistical relationship confirms that usability is a critical factor; when students find the technology easy to navigate and free from undue complexity, it directly encourages its integration into their educational activities. Therefore, the study conclusively establishes that a user-friendly experience is a fundamental prerequisite for fostering the adoption of AI-integrated social media tools in a learning context. Our findings are in line with studies that confirmed PEOU’s positive impact on the adoption intention to use social media for learning^107^.

The significant moderating roles of PU and PEOU underscore that the core drivers of adoption—collaborative learning **(H12)**, social support **(H10 and H13)**, and resource sharing **(H11 and H14)** —are substantially amplified when students find the platform both beneficial and easy to use. This finding has direct implications for design and implementation. A platform’s ease of use—achieved through an intuitive interface, seamless navigation, reliable messaging, and robust privacy controls—lowers the barrier to entry, enabling students to focus on collaborative tasks rather than struggling with the technology. Simultaneously, the platform’s usefulness must be evident through features that yield tangible academic benefits, such as efficient resource sharing, access to diverse information, and the ability to build professional and academic networks. In practice, these insights suggest a dual strategy for boosting adoption. First, institutions should ensure platforms are intuitively designed to maximize PEOU. Second, they should actively demonstrate the PU by integrating the platform into core learning activities and showcasing its value through training and success stories. By strategically enhancing both PU and PEOU, educators can leverage collaborative learning, social support, and resource sharing to their fullest potential, thereby significantly increasing adoption intention.

However, the finding that PU failed to significantly moderate the relationship between collaborative learning and adoption intention **(H9)** is an indication that the motivational drive for students to collaborate with their peers is so potent and intrinsic that it functions independently of the platform’s perceived utility. In essence, the fundamental need to connect, study in groups, and engage in shared academic work is a compelling reason for adoption in its own right. Students appear to view the AI-integrated social media platform primarily as an essential conduit for this collaboration—the digital space where their peers are present. Therefore, while PU is a critical direct driver and even amplifies other factors like resource sharing, its role as a moderator is unnecessary for collaborative learning. The act of collaboration itself is the primary catalyst, suggesting that promotion of these platforms for group work should emphasize their capacity for connection and interaction rather than relying solely on arguments about the specific usefulness of their AI features. These moderating results could not be equated with any past and current literature since they are the novelty and major contribution of this paper.

### Theoretical implications

This study makes several key contributions to the literature by extending and validating the TAM in the context of AI-integrated social media for learning engagement. By integrating the external variables of collaborative learning, social support, resource sharing, and facilitating conditions, the research provides a more nuanced understanding of the drivers behind student adoption intention. The findings offer two primary theoretical advancements/contributions. First, they delineate the distinct moderating roles of the core TAM constructs: while PEOU significantly amplifies the effects of all three social factors (collaborative learning, social support, and resource sharing) on adoption intention, PU only moderates the relationships for social support and resource sharing. Its failure to moderate the link with collaborative learning is a critical distinction, suggesting that the intrinsic motivation to collaborate may supersede utilitarian evaluations. The unique contributions of this study are the validated moderating results of PU and PEOU, moderating the effect of collaborative learning, social support, and resource sharing on AI-integrated social media adoption for learning engagement. These moderated contributions which repositioned TAM cores constructs are differentiated from research works that have also applied TAM in the context of social media adoption, however reports that the use of social media for collaborative learning and student engagement has direct influence on perceived usefulness, ease of use and engagement^28^ and the perceived ease of use drives behavioral intention to engage^108^. More so, a study demonstrated that perceived ease of use and perceived usefulness do not drive social media collaborative learning^109^. Second, the model demonstrates strong explanatory power, with the integrated variables collectively explaining 51.3% of the variance in adoption intention, while facilitating conditions significantly predict both PU and PEOU. It is, however, important to stress that the low R² values of FC impact on PEOU (0.056) and PU (0.046) are a theoretical limitation that deserves attention. This limitation of FC being a poor predictor of PU and PEOU in the context of this study can be explained as FC may be crucial for complicated, new, or costly technologies as compared to ubiquitous technology like the free app of WeChat that students are consistently using, and other facilitating conditions, such as device access, electricity, internet, etc., are guaranteed. Thus, FC acts as a hygiene factor (basic necessity) rather than a “motivator” for perceptions of usefulness or ease of use. It also indicates that the measured variable of FC may have reached the saturation period already. Second, PU and PEOU are internal perceptual beliefs shaped by direct interaction and experience as compared to FC, which is an external, environmental factor. Consequently, the poorer predictive nature of FC in the mature stage of the WeChat context might indicate that internal factors such as user experience with AI features, social influence from peers, and personal innovativeness may be considered more powerful in shaping these perceptions than the external support environment of FC. Thirdly, other possible predictors such as technology literacy, AI self-efficacy and literacy, prior experience with AI, etc., may be the actual drivers of PU and PEOU as compared to FC. Finally, the finding clarifies that for mature social media ecosystems like WeChat, research should shift focus from basic facilitation to the qualitative experience of embedded intelligent features. These results provide a validated, extended TAM framework that scholars can apply to further investigate the adoption of AI-integrated social media, both in educational settings and other technological contexts.

## Conclusion

Based on an expanded Technology Acceptance Model (TAM), this study explores the key factors influencing students’ adoption of AI-integrated WeChat Social Media to enhance their learning engagement. Using a PLS SEM approach, we found that collaborative learning, social support, resource sharing, and facilitating conditions all significantly and positively impacted adoption intention. Furthermore, facilitating conditions were shown to directly influence the core TAM variables, i.e., PU and PEOU. Moderation analysis further revealed that PEOU significantly enhanced the positive impact of three social factors (collaborative learning, social support, and resource sharing) on ​​adoption intention, providing a deeper explanation for the utility boundaries of social motivations. In contrast, PU only moderated the relationship between social support, resource sharing, and adoption intention, and did not significantly influence the association between collaborative learning and adoption intention. This suggests that students’ intrinsic motivation for collaboration is sufficiently strong that it does not require a higher evaluation of the tool’s usefulness. These findings further highlight the importance for universities and educational stakeholders to organically integrate AI-integrated social media into the teaching and learning ecosystem, prioritizing strategies that enhance usability and improve enabling conditions. To fully unleash the educational potential of AI-powered social media, schools must proactively coordinate and guide initiatives, focusing on three key areas: (a) Academic Benefits: collaborative, deep learning enhances critical thinking, problem-solving, and classroom performance; (b) Psychological Benefits: Student-led collaborative activities enhance self-esteem, alleviate anxiety, and foster positive attitudes; (c) Social Benefits: building supportive learning communities within collaborative digital environments and demonstrating effective collaboration. Through these ways, educational institutions can significantly enrich the learning experience and comprehensively improve academic outcomes.

## Limitations and future research

First, the findings are grounded in the Chinese social media context, which is dominated by platforms like WeChat. Consequently, the results may not be directly generalizable to other cultural or technological ecosystems, and therefore, cross-validation in different national contexts is recommended. Second, the use of cross-sectional data provides a snapshot in time but cannot capture the evolution of user behavior. Future research would benefit from a longitudinal design to track how adoption intentions translate into sustained usage and how perceptions change over time. Finally, while this study identified several key drivers, it does not encompass all potential factors influencing adoption. Future investigations could incorporate additional variables such as perceived enjoyment, social influence, digital literacy, and technology self-efficacy to develop a more comprehensive model. Examining whether these factors mediate the relationship between the social drivers (collaborative learning, social support, resource sharing) and adoption intention would be a particularly valuable contribution. Additionally, technology literacy, AI self-efficacy, and AI literacy, and prior experience with AI could be integrated into the model for future testing to confirm if it can better predict or account for more variance in PU and PEU, respectively, than FC has demonstrated in this study.

## Data Availability

The raw data will be made available upon reasonable request to the corresponding author.
